# High-Intensity Post-Stroke Rehabilitation Is Associated with Lower Risk of Pressure Ulcer Development in Patients with Stroke: Real-World Evidence from a Nationwide, Population-Based Cohort Study

**DOI:** 10.3390/medicina58030402

**Published:** 2022-03-08

**Authors:** Ying-Chu Chen, Tai-Li Chen, Chia-Chun Cheng, Yu-Cih Yang, Jen-Hung Wang, Hei-Tung Yip, Chung-Yi Hsu, Hung-Yu Cheng

**Affiliations:** 1 Buddhist Tzu Chi Medical Foundation, Department of Physical Medicine and Rehabilitation, Hualien Tzu Chi Hospital, Tzu Chi University, Hualien 970, Taiwan; cityofstars1497@gmail.com; 2 Buddhist Tzu Chi Medical Foundation, Center for Aging and Health, Hualien Tzu Chi Hospital, Hualien 970, Taiwan; terrychen.a@gmail.com; 3 Department of Dermatology, Taipei Veterans General Hospital, Taipei 112, Taiwan; 4 Buddhist Tzu Chi Medical Foundation, Department of Medical Education, Medical Administration Office, Hualien Tzu Chi Hospital, Hualien 970, Taiwan; 5 Buddhist Tzu Chi Medical Foundation, Department of Pathology, Hualien Tzu Chi Hospital, Tzu Chi University, Hualien 970, Taiwan; 99311117@gms.tcu.edu.tw; 6 Management Office for Health Data, China Medical University Hospital, Taichung 404, Taiwan; moiluvkiwi@gmail.com (Y.-C.Y.); fionyip0i0@gmail.com (H.-T.Y.); 7 College of Medicine, China Medical University, Taichung 404, Taiwan; 8 Buddhist Tzu Chi Medical Foundation, Department of Medical Research, Hualien Tzu Chi Hospital, Tzu Chi University, Hualien 970, Taiwan; jenhungwang2011@gmail.com; 9 Graduate Institute of Clinical Medical Science, China Medical University, Taichung 404, Taiwan; hsuc@mail.cmu.edu.tw

**Keywords:** pressure ulcers, pressure injuries, stroke, rehabilitation

## Abstract

*Background and Objectives:* Multiple factors are associated with pressure ulcer (PU) development, including limited mobility following stroke. We performed a nationwide cohort study to investigate the impact of rehabilitation intensity on the incidence of post-stroke PU. *Materials and Methods:* Data of patients diagnosed with stroke between 2000 and 2012 were collected from the 2000 Longitudinal Health Insurance Database (Taiwan). Based on the number of rehabilitation sessions attended within 90 days of discharge, the rehabilitation intensity was classified as low, medium, or high. After adjusting for sociodemographic factors and comorbidities, the Cox proportional hazards model evaluated the risk of PU development during the 12-year follow-up period. Kaplan–Meier curves were used to estimate the cumulative incidence of PUs. *Results:* Our study included 18,971 patients who had their first episode of stroke. Of these, 9829 (51.8%) underwent rehabilitation therapy after discharge. Female patients and patients with a National Institutes of Health Stroke Scale (NIHSS) score >13 points, who commenced high-intensity post-stroke rehabilitation after discharge had a significantly lower risk of PU development than those who underwent low-intensity post-stroke rehabilitation after discharge. Cumulative survival analysis showed a significantly lower cumulative incidence of PU during the 12-year follow-up period in the high-intensity rehabilitation group. *Conclusion:* Compared with low-intensity post-stroke rehabilitation, high-intensity post-stroke rehabilitation after discharge from hospital is associated with a lower risk of post-stroke PU development, especially in female stroke patients and patients with a NIHSS score >13 points. High-intensity rehabilitation is also associated with a significantly lower cumulative incidence of PU events during the 12-year follow-up period.

## 1. Introduction

Pressure ulcers (PU) are a worldwide health problem occurring in 50% of critically ill patients and in more than 70% of elderly patients in nursing homes in the United States [1]; it occurs commonly in stroke patients, diminishing their quality of life and significantly increasing morbidity and mortality [2,3]. During hospitalization in a stroke-specific Indonesian hospital, 22% of stroke patients were found to have PUs [4]; other studies showed that the frequency of PUs after stroke ranges from 0.7% in a rehabilitation setting in Singapore to 18% in an acute hospital setting in Scotland [5]. In addition, PU-related bacteremia and infections are potential contributors to post-stroke complications and mortality [1]. It is estimated that 65,000 out of every 1 million patients who develop PUs die from complications; this presents a major health problem worldwide [6].

Doubtlessly, stroke remains a leading cause of mortality and disability worldwide [7], with an estimated 16 million people affected annually [8]. Post-stroke rehabilitation requires a sustained and coordinated effort from a large team, including the patients striving for their goals, family and friends, major caregivers, physicians, nurses, physical, occupational, speech, and recreational therapists, psychologists, nutritionists, social workers, and others [9]. With cognitive, functional, and sensory deficits after a stroke, patients are at a higher risk of complications, which result in poor functional outcomes [10]. Immobility-related complications are common among stroke survivors in the first year after a severely disabling stroke [10], with half of them unable to return to work because of post-stroke disabilities [11,12].

To the best of our knowledge, more than two-thirds of stroke survivors receive rehabilitation services after hospitalization [9], with evidence suggesting that stroke rehabilitation may help improve motor function [13]. However, there is little evidence of the association between rehabilitation and the development of PUs in post-stroke patients after discharge from the hospital. Based on data from the Taiwan National Health Insurance Research Database (NHIRD), we conducted a nationwide cohort study to investigate the impact of rehabilitation intensity on the presence of PUs after stroke.

## 2. Materials and Methods

### 2.1. Study Design and Data Sources

In this nationwide, population-based cohort study, we retrieved data from the 2000 Longitudinal Health Insurance Database (LHID 2000) [14,15]. Under the Universal Health Coverage Project, data were collected from virtually all healthcare services, including medical facility registries, medication prescriptions, and outpatient, inpatient, and emergency visiting data for Taiwan’s general public. The LHID 2000 consisted of 1 million people randomly selected from the NHIRD (which recruited more than 99% of the inhabitants in Taiwan). Since previous studies found no statistically significant difference in age, sex, medication use, and disease diagnosis between the LHID sample cohort and the registry of all NHI beneficiaries, the representativeness of the LHID is validated. More than 20,000 medical care facilities, including hospitals, clinics, and pharmacies, which represent over 93% of all healthcare facilities in Taiwan, were contracted by the National Health Insurance (NHI) project. All populations were followed up for outcome identification using the International Classification of Diseases, Ninth Revision, Clinical Modification (ICD-9-CM) codes. This study was approved by the Institutional Review Board (IRB) of the China Medical University and Hospital Research Ethics Committee (IRB: CMUH-104-REC2-115). The need for informed consent was waived due to the encryption of LHID.

### 2.2. Study Population

**Figure 1:**
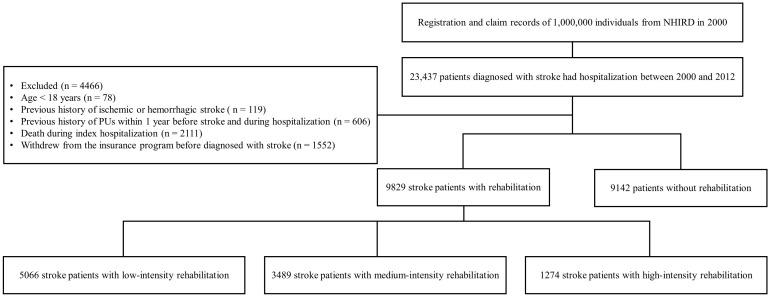
Flow chart illustrating the selection process of study subjects from the National Health Insurance Research Database (NHIRD) in Taiwan.

Individuals aged ≥18 years who had been diagnosed with stroke were enrolled in our study using ICD-9-CM codes 430–434, 436, or 438. The concurrent hospitalization was recorded as the index hospitalization. The first day after discharge from the hospital following the latest stroke episode was recorded as the index date. Only patients with an inpatient diagnosis of stroke before the index date were recruited to avoid overestimation. Patients who fulfilled the following criteria were excluded from this study: (1) age < 18 years; (2) previous history of ischemic or hemorrhagic stroke; (3) previous history of PUs within 1 year prior to stroke and during hospitalization; (4) death during index hospitalization; (5) withdrew from the insurance program before diagnosed with stroke. These codes were used in previous epidemiologic studies that employed the LHID [16,17,18] (Figure 1).

To evaluate the association between rehabilitation intensity and PUs in stroke patients, the enrollees were divided into two groups. We, therefore, established a rehabilitation and non-rehabilitation group. The rehabilitation group included patients undergoing post-stroke rehabilitation, including physical, occupational, and speech therapies, according to the NHI medical records within 90 days of being discharged from the hospital. The rehabilitation group was further categorized according to rehabilitation intensity into low-, medium-, and high-intensity groups that received 1–3, 4–14, and ≥15 therapy sessions, respectively [19].

### 2.3. Outcome

The primary outcome of this study was the incidence of PUs (ICD-9-CM 707.x). The diagnosis of PU was defined as either a discharge diagnosis of PU or a diagnosis of PU confirmed at least twice in an outpatient department by plastic surgeons. These codes were used in previous epidemiologic studies [1,20,21,22]. All subjects were followed up from the index date until 31 December 2012, a new diagnosis of PU, or withdrawal from the NHI program.

### 2.4. Covariates

Potential confounding factors were selected from previous epidemiologic studies, such as comorbid medical illnesses, stroke type, Charlson Comorbidity Index (CCI), National Institutes of Health Stroke Scale (NIHSS) score, hospitalization level, urbanization of residence, and residential area. Comorbid medical illnesses included hypertension (ICD-9-CM codes 401–405), diabetes mellitus (ICD-9-CM code 250), coronary heart disease (ICD-9-CM codes 410–414), congestive heart failure (ICD-9-CM code 428), hyperlipidemia (ICD-9 CM codes 272.0–272.4), peripheral artery disease (ICD-9-CM codes 440–449), obesity (ICD-9-CM code 278), malnutrition (ICD-9-CM codes 263, 764), Parkinson’s disease (ICD-9-CM code 332.0), dementia (ICD-9-CM codes 294.1, 294.10, 294.11, 294.2, 294.20, 294.21), depression (ICD-9-CM codes 296, 309, 311), malignancy (ICD-9-CM codes 140–208), and hemiplegia (ICD-9-CM code 342.x). We also used an estimated NIHSS score to define the severity of stroke based on the Stroke Severity Index (SSI) during index hospitalization. The claims-based SSI developed by Sung et al. is highly correlated with the NIHSS; it can be converted to the NIHSS for patients with ischemic stroke as follows: estimated NIHSS = 1.1722 × SSI − 0.7533 [23]. Furthermore, the CCI score (0, 1, or >2) was calculated using participant status before the index date. 

### 2.5. Statistical Analysis

Distribution comparisons of categorical variables in the baseline characteristics, such as age stratification, sex, stroke type, CCI, NIHSS score, hospital level, residential area, and comorbidities, were performed via univariate analysis with chi-square testing. Continuous variables, such as average age and length of hospital stay, were analyzed using Student’s *t*-test to compare the difference between the rehabilitation and comparison cohorts. The subjects were classified into four regions in Taiwan (northern, central, eastern, and southern) to distinguish the geographic location. The urbanization level was divided by where they lived based on NHIRD information into four levels, in which level 1 was the most urbanized and level 4 was the least urbanized. The classification criteria included population density (persons per km^2^), percentage of people with college-level education or higher, percentage of the elderly (older than 65 years), and the number of physicians per 100,000 population [24,25]. Multivariate Cox proportional hazard regression models adjusted for age, sex, stroke type, CCI, NIHSS score, hospital level, residential area, and comorbidities were developed to assess the risk of PUs in the rehabilitation group compared with that in the non-rehabilitation group. We also used Cox regression analyses to compare the risk of PUs among the rehabilitation intensity groups. The Kaplan–Meier method and the log-rank test were used to estimate the difference in cumulative incidence of PUs between the two groups. All statistical analyses were performed using SAS statistical software, version 9.4 for Windows (SAS Institute, Inc., Cary, NC, USA).

## 3. Results

The selection process is illustrated in Figure 1. In our study, a total of 23,437 stroke patients were identified from the national cohort. After excluding patients not meeting study criteria, 18,971 patients were investigated. The rehabilitation group consisted of 9829 patients, while the comparison cohort consisted of 9142 patients. The baseline characteristics of the two cohorts are presented in Table 1. The mean age of the rehabilitation and non-rehabilitation groups was 69.0 and 69.4 years, respectively. The dominant characteristics were: male patients, age more than 65 years, NIHSS score less than 13, urbanization level 4, and patients living in the northern region.

Patients undergoing rehabilitation demonstrated a higher prevalence of hypertension, diabetes mellitus, and hemiplegia and a lower prevalence of coronary heart disease, congestive heart failure, peripheral artery disease, malnutrition, depression, and malignancy.

A total of 1080 patients with new-onset PUs after stroke were identified in the national cohort (Figure 1). As shown in Table 2 and Table 3, the overall incidence of PUs was 2.42, 2.4, and 1.76 per 100 person-years in the low-, medium-, and high-intensity rehabilitation groups, respectively. The risk of PU remained significant after adjusting for potential confounders in the low- and medium-intensity rehabilitation groups (adjusted HR = 1.27, 95% CI = 1.13–1.41; adjusted HR = 1.21, 95% CI = 1.07–1.37, respectively).

Compared with the non-rehabilitation group, the low- and medium-intensity rehabilitation groups had higher risks of PU in men (adjusted HR = 1.35, 95% CI = 1.16–1.56; adjusted HR = 1.26, 95% CI = 1.07–1.49, respectively), patients older than 65 years (adjusted HR = 1.26, 95% CI = 1.11–1.43; adjusted HR = 1.17, 95% CI = 1.02–1.35), CCI where the score is 1 (adjusted HR = 1.42, 95% CI = 1.18–1.71; adjusted HR = 1.32, 95% CI = 1.08–1.62, respectively), patients with NIHSS score ≤13 (adjusted HR = 1.32, 95% CI = 1.16–1.51; adjusted HR = 1.38, 95% CI = 1.19–1.60), community hospital level (adjusted HR = 1.25, 95% CI = 1.03–1.50; adjusted HR = 1.31, 95% CI = 1.06–1.62), patients with hypertension (adjusted HR = 1.25, 95% CI = 1.17–1.41; adjusted HR = 1.16, 95% CI = 1.01–1.32, respectively), patients with diabetes mellitus (adjusted HR = 1.21, 95% CI = 1.03–1.41; adjusted HR = 1.21, 95% CI = 1.02–1.44, respectively), patients with coronary heart disease (adjusted HR = 1.29, 95% CI = 1.10–1.53; adjusted HR = 1.27, 95% CI = 1.05–1.54), and patients with hyperlipidemia (adjusted HR = 1.39, 95% CI = 1.16–1.67; adjusted HR = 1.28, 95% CI = 1.05–1.57). Compared with the non-rehabilitation group, the high-intensity rehabilitation group had a lower risk of PU, with an NIHSS score >13 (adjusted HR = 0.71, 95% CI = 0.50–0.99).

Table 4 presents the incidence and HR of PUs among the three rehabilitation intensity groups. Compared with the low-intensity rehabilitation group, the high-intensity rehabilitation group had a lower risk of PU in women (adjusted HR = 0.67, 95% CI = 0.48–0.94) and in those with an NIHSS score >13 (adjusted HR = 0.63, 95% CI = 0.45–0.88). 

**Figure 2:**
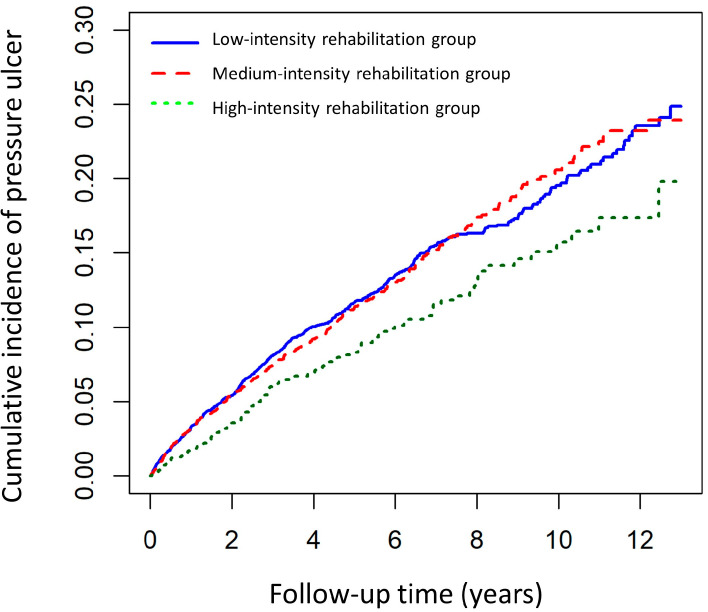
Using Kaplan–Meier survival statistics, it showed crude overall survival curves with rehabilitation intensity stratification (log-rank *p* = 0.007).

Figure 2 shows the Kaplan–Meier curve for cumulative PU risk stratified by rehabilitation intensity using a log-rank test. The high-intensity rehabilitation group was associated with a significantly decreased risk of PU events (log-rank *p* = 0.007). From the first year of follow-up to the end of the study period, the incidence of PU events in the high-intensity rehabilitation group was consistently lower than that in the medium- and low-intensity rehabilitation groups.

## 4. Discussion

In this study, we analyzed real-world data to investigate the association between post-stroke rehabilitation intensity and the risk of PU development. We observed that patients undergoing high-intensity rehabilitation had a lower cumulative incidence of PUs. To the best of our knowledge, this is the first large-scale study to demonstrate a reduced risk of PUs in patients with stroke who received high-intensity rehabilitation.

We found that the risk of developing PU was higher in the low- and medium-intensity rehabilitation groups than in the high-intensity group. We speculate that rehabilitation has cumulative effects against PU prevention and that there is a minimum rehabilitation intensity required for effective PU prevention. Previous studies reported that immobility was significantly associated with the risk of PUs [26] and that the Functional Independence Measure score at admission was strongly correlated with PU development [27]. This suggests that the most important benefits of post-stroke rehabilitation include increased mobility and improved functional recovery.

A significantly lower risk of PU development was observed in patients with NIHSS scores >13 points in the high-intensity group; this implies that there are benefits of rehabilitation training programs for patients with severe stroke who require long-term, skilled care [26]. In addition, several guidelines suggest early mobilization and rehabilitation after stroke to obtain beneficial effects [28,29]. After a stroke episode, the window of opportunity for brain plasticity and repair is limited, making early neurological rehabilitation optimal for stroke recovery [30]. In terms of sex-based differences, women who had an ischemic stroke had better survival rates but were also more disabled and had a poorer quality of life than their male counterparts [31]. In Taiwanese families, women are regarded as the most appropriate caregivers for all family members [32]. The increasing demand for elderly care in Taiwan and the changing family structures and competing roles for women have directly influenced the health of the caregivers and care-receivers [33]. If female family members experience a stroke event, their family’s equilibrium is disrupted; this affects their overall quality of life [34]. In our study, we noted that, in the high-intensity rehabilitation group, women were at a lower risk of developing PU. One possible explanation is that the cumulative effect of rehabilitation training offers protection against PU formation, especially to women who have the opportunity to receive high-intensity rehabilitation; this may be associated with a better family supportive care system. In addition, according to the Taiwanese family culture mentioned above, if men experience a stroke, women in the family tend to be the most appropriate caregivers; this may reduce the variability of care providers for men with stroke compared to that for women with stroke. Overall, we believe that high-intensity rehabilitation for women with stroke may exert a better protective effect against PU formation than low- or medium-intensity rehabilitation; this may be due to better family supportive care, which gives them the opportunity to receive rehabilitation training.

Our cumulative survival analysis showed a significantly lower cumulative incidence of PU during the 12-year follow-up period in the high-intensity rehabilitation group. Community- and home-based rehabilitation programs have various benefits, including reduced costs, decreased length of stay in hospitals or institutional settings, more opportunity for involvement of patients and their family in the treatment process, and less stress on caregivers and family members [35,36]. Some community-based rehabilitation intervention trials demonstrated enhanced ambulation and mobility, better self-care, and greater functional independence [37]. There is also substantial evidence that rehabilitation services, especially exercise-based programs provided in the community after discharge from acute or institutional care, can improve cardiovascular health and decrease the risk of cardiovascular events, leading to increased short-term survival rates for individuals who have experienced a stroke [9]. The studies above reveal how vital the mobility status of post-stroke patients is and how largely their lifestyle (including rehabilitation intervention) influences their long-term prognosis.

Aside from rehabilitation intensity, there are other important factors associated with the presence of PUs. A previous study reported that hypoalbuminemia increases the likelihood of developing pneumonia, other infections, and gastrointestinal bleeding after hospitalization for stroke in patients with PUs compared to patients without PU who have hypoalbuminemia [1]. Diabetes mellitus is also a risk factor for the development of PUs in patients with ischemic stroke [38]. With long-term blood glucose abnormalities, the effect of diabetes mellitus on PUs may be associated with diabetic neuropathy and vascular pathologies. Moreover, the level of consciousness in patients who have developed PUs may deteriorate with stroke, making it more difficult for them to express their symptoms or communicate with medical caregivers [1].

Previous studies showed that the rate of rehabilitation utilization was approximately 50% in ischemic stroke patients in Taiwan [39,40]. Yeh et al. surveyed long-term patterns of rehabilitation utilization among patients with stroke in Taiwan. They reported that 44.7% and 33.8% of these patients received physical and occupational therapy, respectively, over a two-year period [27]. The proportion of rehabilitation recipients among all patients with stroke in our study was 51.8%. In particular, patients with hemiplegia and other comorbidities utilized rehabilitation programs more than those without. A possible explanation is that patients with mild stroke and few comorbidities are more likely to have better prognosis and recovery, leading them to demand for rehabilitation less. 

The present study has several strengths. First, the results were based on real-world evidence of daily clinical practice from a nationwide, population-based database. Second, we believe that our study findings contribute significantly to the literature, as we showed that high-intensity rehabilitation training has a potentially positive impact on stroke patients by lowering their risk of developing PU. Further studies may establish effective interventions with rehabilitation training to prevent PUs after stroke. 

The present study has several limitations. First, we could only elucidate the association but not the actual causality between rehabilitation intensity and the incidence of PU. Further clinical trials are warranted. Second, the number of patients with PU may have been underestimated, though the accuracy of the diagnostic codes has been verified in previous studies. Patients with less severe or early-stage PU may not seek medical aid; therefore, patients with mild PU may not be registered in our cohort. Finally, we could not retrieve details on post-stroke rehabilitation prescriptions, including the frequency and duration of training programs, progressive adjustment of goals, and composition of any therapies from the LHID 2000. Taiwan’s national health insurance provides fixed cash payments for post-stroke rehabilitation, including one session of physical therapy, occupational therapy, or speech therapy, respectively, per day. Each therapy session has a minimum duration of at least 50 min, but no ceiling duration is applied. Therefore, we could only retrieve the number of whole sessions rather than the actual therapy duration of post-stroke rehabilitation from the LHID. Future, in-depth research on the impact of rehabilitation on PU risk in stroke patients may be helpful.

## 5. Conclusions

In summary, our study demonstrated that, compared with low-intensity post-stroke rehabilitation, high-intensity post-stroke rehabilitation after discharge from hospital is associated with a significantly lower risk of PU development, especially in female stroke patients and patients with a NIHSS score >13 points. High-intensity rehabilitation is also associated with a significantly lower cumulative incidence of PU events during the 12-year follow-up period. Further investigations are needed to confirm this finding.

## Figures and Tables

**Table 1:** Baseline characteristics and comorbidity of patients with and without post-stroke rehabilitation.

	Rehabilitation		Non-Rehabilitation		*p*-Value *
	(*n* = 9829)		(*n* = 9142)		
	*n*	%	*n*	%	
Age					0.95
<65	3523	38.5	3784	38.5	
≥65	5619	61.5	6045	61.5	
Sex					0.02
Female	3981	40.5	3554	38.9	
Male	5848	59.5	5588	61.1	
Median (IQR) ^†^	69.0 (58.8, 77.0)		69.4 (58.6, 77.5)		0.26
Stroke type					<0.0001
Ischemic stroke	7909	80.5	7945	86.9	
Intracerebral stroke	1920	19.5	1197	13.1	
Rehabilitation intensity					
1–3 Times	5066	51.5	-	-	
4–14 Times	3489	35.5	-	-	
≥15 Times	1274	13	-	-	
Charlson comorbidity index					<0.0001
0	2572	26.2	3410	37.3	
1	2934	29.8	2456	26.9	
≥2	4323	44	3276	35.8	
Comorbidity					
Hypertension	8527	86.8	7546	82.5	<0.0001
Diabetes mellitus	4201	42.7	3660	40	0.0002
Coronary heart disease	3750	38.1	3929	43	<0.0001
Congestive heart failure	1676	17.1	1716	18.8	0.002
Hyperlipidemia	4102	41.7	3741	40.9	0.25
Peripheral artery disease	1060	10.8	1192	13	<0.0001
Obesity	96	0.98	86	0.94	0.79
Malnutrition	67	0.68	89	0.97	0.02
Parkinson’s disease	345	3.51	352	3.85	0.21
Dementia	302	3.07	319	3.49	0.1
Depression	843	8.58	923	10.1	0.0003
Malignancy	925	9.41	1019	11.1	<0.0001
Hemiplegia	136	1.38	68	0.74	<0.0001
Length of hospital stay (days)					<0.0001
Median (IQR) ^†^	52 (28, 73)		38 (26, 57)		
NIHSS score					<0.0001
≤13	7052	71.8	7754	84.8	
>13	2777	28.2	1388	15.2	
Hospital level					<0.0001
Medical center	2930	29.8	2456	26.9	
Regional hospital	4096	41.7	3754	41.1	
Community hospital	2803	28.5	2932	32	
Urbanization level					0.15
1 (highest)	2405	24.5	2292	25.1	
2	2746	27.9	2472	27	
3	1704	17.3	1517	16.6	
4 (lowest)	2974	30.3	2861	31.3	
Residential area of Taiwan					0.12
Northern	3713	37.8	3469	37.9	
Central	2156	21.9	1916	21	
Southern	2860	29.1	2778	30.4	
Eastern	1100	11.2	979	10.7	
Death	1345	13.7	1167	12.8	0.06

NIHSS: National Institute of Health Stroke Scale; * *p*-value using chi-square for the comparisons between with and without rehabilitation; ^†^ Average age and length of hospital stay using Wilcoxon sum rank test for verification; The urbanization level was categorized by the population density of the residential area into 4 levels, with level 1 as the most urbanized and level 4 as the least.

**Table 2:** Incidence rate and hazard ratio of pressure ulcer among four groups stratified by age, sex, Charlson comorbidity index, NIHSS score, hospital level, urbanization level, and residential area of Taiwan.

	Rehabilitation Intensity														
	No (*n* = 9142)			1–3 Times (*n* = 5066)			Adjusted HR ^†^ (95%CI)	4–14 Times (*n* = 3489)			Adjusted HR ^†^ (95%CI)	≥15 Times (*n* = 1274)			Adjusted HR ^†^ (95%CI)
	Event	PY	IR	Event	PY	IR		Event	PY	IR		Event	PY	IR	
Overall															
Pressure ulcer	770	46,973	1.64	567	23,404	2.42	1.27 (1.13–1.41) ***	401	16,691	2.4	1.21 (1.07–1.37) **	112	6374	1.76	0.95 (0.78–1.17)
Age															
<65	160	21,649	0.74	113	10,147	1.11	1.27 (0.99–1.62)	97	7820	1.24	1.19 (0.91–1.54)	41	3270	1.25	1.14 (0.80–1.63)
≥65	610	25,324	2.41	454	13,257	3.42	1.26 (1.11–1.43) ***	304	8871	3.43	1.17 (1.02–1.35) *	71	3104	2.29	0.81 (0.63–1.04)
Sex															
Female	350	18,858	1.86	247	9517	2.6	1.18 (1.00–1.40) *	166	6684	2.48	1.16 (0.96–1.41)	41	2739	1.5	0.74 (0.53–1.04)
Male	420	28,115	1.49	320	13,887	2.3	1.35 (1.16–1.56) ***	235	10,007	2.35	1.26 (1.07–1.49) **	71	3635	1.95	1.14 (0.88–1.47)
Charlson comorbidity index															
0	127	19,286	0.66	65	7164	0.91	1.36 (1.00–1.84) *	36	3813	0.94	1.44 (0.98–2.12)	10	1526	0.66	1.07 (0.55–2.06)
1	253	13,559	1.87	215	7235	2.97	1.42 (1.18–1.71) ***	161	5750	2.8	1.32 (1.08–1.62) **	39	2016	1.93	1.00 (0.71–1.41)
≥2	390	14,128	2.76	287	9005	3.19	1.14 (0.98–1.33)	204	7128	2.86	1.09 (0.91–1.30)	63	2832	2.22	0.90 (0.68–1.18)
NIHSS score															
≤13	588	40,608	1.45	389	19,033	2.04	1.32 (1.16–1.51) ***	247	11,178	2.21	1.38 (1.19–1.60) ***	68	4195	1.62	1.09 (0.84–1.40)
>13	182	5365	3.39	178	4371	4.07	1.12 (0.91–1.39)	154	5513	2.79	0.91 (0.73–1.14)	44	2179	2.02	0.71 (0.50–0.99) *
Hospital level															
Medical center	158	12,985	1.22	170	7816	2.18	1.44 (1.16–1.80) **	107	5053	2.12	1.20 (0.94–1.55)	30	1718	1.75	1.13 (0.76–1.69)
Regional hospital	310	19,810	1.56	212	9376	2.26	1.19 (1.00–1.42) *	155	7023	2.21	1.13 (0.92–1.38)	50	2924	1.71	0.92 (0.67–1.24)
Community hospital	302	14,178	2.13	185	6212	2.98	1.25 (1.03–1.50) *	139	4615	3.01	1.31 (1.06–1.62) **	32	1732	1.85	0.86 (0.59–1.25)
Urbanization level															
1 (highest)	165	11,871	1.39	132	5672	2.33	1.40 (1.11–1.77) **	86	4541	1.89	1.10 (0.84–1.44)	19	1387	1.37	0.91 (0.56–1.47)
2	195	12,837	1.52	134	6158	2.18	1.17 (0.94–1.47)	105	4773	2.2	1.12 (0.87–1.43)	40	1995	2.01	1.04 (0.73–1.48)
3	127	7887	1.61	103	4370	2.36	1.31 (1.00–1.72) *	61	2614	2.33	1.24 (0.91–1.71)	15	1141	1.31	0.73 (0.42–1.25)
4 (lowest)	283	14,352	1.97	198	7180	2.76	1.26 (1.05–1.51) *	149	4749	3.14	1.37 (1.11–1.68) **	38	1851	2.05	1.02 (0.72–1.45)
Residential area of Taiwan															
Northern	264	17,902	1.47	211	9039	2.33	1.36 (1.13–1.64) ***	138	6774	2.04	1.10 (0.89–1.36)	29	2115	1.37	0.85 (0.57–1.25)
Central	173	10,016	1.73	105	5059	2.08	1.07 (0.84–1.37)	106	3367	3.15	1.56 (1.21–2.01) ***	35	1520	2.3	1.14 (0.78–1.65)
Southern	240	14,151	1.7	169	6866	2.46	1.23 (1.00–1.50) *	99	4640	2.13	1.07 (0.84–1.37)	35	2045	1.71	0.90 (0.62–1.29)
Eastern	93	4904	1.9	82	2440	3.36	1.57 (1.16–2.14) **	58	1910	3.04	1.36 (0.96–1.93)	13	694	1.87	0.92 (0.49–1.71)

PY, person-years; IR, incidence rate per 100 person-years; HR, hazard ratio; CI, confidence interval; ^†^ HR adjusted for age, sex, comorbidities, NIHSS score, hospital level, urbanization level, and residential area of Taiwan; * *p* < 0.05, ** *p* < 0.01, *** *p* < 0.001.

**Table 3:** Incidence rate and hazard ratio of pressure ulcer among four groups stratified by comorbidities.

	Rehabilitation Intensity														
	No (*n* = 9142)			1–3 Times (*n* = 5066)			Adjusted HR ^†^ (95%CI)	4–14 Times (*n* = 3489)			Adjusted HR ^†^ (95%CI)	≥15 Times (*n* = 1274)			Adjusted HR ^†^ (95%CI)
	Event	PY	IR	Event	PY	IR		Event	PY	IR		Event	PY	IR	
Comorbidity															
Hypertension															
No	99	9337	1.06	63	3286	1.92	1.35 (0.97–1.86)	49	2397	2.04	1.72 (1.20–2.46) **	11	813	1.35	1.21 (0.64–2.30)
Yes	671	37,636	1.78	504	20,118	2.51	1.25 (1.17–1.41) ***	352	14,294	2.46	1.16 (1.01–1.32) *	101	5561	1.82	0.92 (0.74–1.14)
Diabetes mellitus															
No	371	29,867	1.24	285	14,137	2.02	1.34 (1.15–1.57) ***	190	10,389	1.83	1.25 (1.04–1.50) *	60	3891	1.54	1.11 (0.84–1.47)
Yes	399	17,106	2.33	282	9267	3.04	1.21 (1.03–1.41) *	211	6302	3.35	1.21 (1.02–1.44) *	52	2483	2.09	0.84 (0.63–1.13)
Coronary heart disease															
No	407	28,599	1.42	328	15,043	2.18	1.24 (1.07–1.43) **	235	10,975	2.14	1.19 (1.01–1.41) *	75	4390	1.71	1.07 (0.83–1.38)
Yes	363	18,374	1.98	239	8361	2.86	1.29 (1.10–1.53) **	166	5716	2.9	1.27 (1.05–1.54) *	37	1984	1.86	0.79 (0.56–1.11)
Congestive heart failure															
No	582	40,519	1.44	460	20,072	2.29	1.35 (1.19–1.52) ***	321	14,599	2.2	1.25 (1.09–1.44) **	95	5636	1.69	1.02 (0.82–1.27)
Yes	188	6454	2.91	107	3332	3.21	1.03 (0.80–1.31)	80	2092	3.82	1.10 (0.84–1.45)	17	738	2.3	0.70 (0.42–1.17)
Hyperlipidemia															
No	497	28,625	1.74	352	14,272	2.47	1.21 (1.06–1.39) **	249	10,266	2.43	1.19 (1.02–1.39) *	75	3954	1.9	1.01 (0.79–1.30)
Yes	273	18,348	1.49	215	9132	2.35	1.39 (1.16–1.67) ***	152	6425	2.37	1.28 (1.05–1.57) *	37	2420	1.53	0.86 (0.61–1.22)
Peripheral artery disease															
No	654	41,900	1.56	493	21,196	2.33	1.26 (1.12–1.42) ***	345	15,238	2.26	1.18 (1.03–1.35) *	96	5913	1.62	0.92 (0.74–1.14)
Yes	116	5073	2.29	74	2208	3.35	1.31 (0.97–1.76)	56	1453	3.85	1.45 (1.04–2.02) *	16	461	3.47	1.21 (0.71–2.07)
Obesity															
No	763	46,589	1.64	564	23,204	2.43	1.27 (1.14–1.42) ***	400	16,539	2.42	1.22 (1.08–1.39) **	112	6323	1.77	0.96 (0.78–1.17)
Yes	7	384	1.82	3	200	1.5	–	1	152	0.66	–	0	51	0	–
Malnutrition															
No	755	46,706	1.62	561	23,270	2.41	1.27 (1.14–1.42) ***	398	16,622	2.39	1.22 (1.07–1.38) **	112	6361	1.76	0.96 (0.78–1.17)
Yes	15	267	5.62	6	134	4.48	1.17 (0.34–3.92)	3	69	4.35	1.39 (0.29–6.73)	0	13	0	–
Parkinson’s disease															
No	711	45,541	1.56	526	22,760	2.31	1.25 (1.12–1.41) ***	374	16,282	2.3	1.20 (1.05–1.37) **	106	6258	1.69	0.94 (0.76–1.16)
Yes	59	1432	4.12	41	644	6.37	1.54 (1.00–2.38) *	27	409	6.6	1.41 (0.86–2.29)	6	116	5.17	1.25 (0.52–3.01)
Dementia															
No	718	45,941	1.56	528	2843	18.6	1.27 (1.13–1.43) ***	386	16,374	2.36	1.22 (1.07–1.39) **	109	6273	1.74	0.96 (0.78–1.17)
Yes	52	1032	5.04	39	561	6.95	1.26 (0.81–1.94)	15	317	4.73	1.02 (0.56–1.86)	3	101	2.97	0.67 (0.20–2.27)
Depression															
No	685	42,683	1.6	510	21,619	2.36	1.25 (1.12–1.41) ***	359	15,424	2.33	1.17 (1.03–1.34) *	103	5846	1.76	0.96 (0.78–1.18)
Yes	85	4290	1.98	57	1785	3.19	1.38 (0.98–1.95)	42	1267	3.31	1.67 (1.13–2.47) **	9	528	1.7	0.91 (0.45–1.48)
Malignancy															
No	679	42,983	1.58	511	21,750	2.35	1.26 (1.12–1.41) ***	380	15,605	2.44	1.24 (1.09–1.42) ***	99	5938	1.67	0.91 (0.74–1.13)
Yes	91	3990	2.28	56	1654	3.39	1.31 (0.94–1.84)	21	1086	1.93	0.74 (0.45–1.21)	13	436	2.98	1.27 (0.70–2.30)
Hemiplegia															
No	764	46,718	1.64	559	23,206	2.41	1.26 (1.13–1.40) ***	397	16,434	2.42	1.22 (1.07–1.38) **	110	6200	1.77	0.97 (0.79–1.18)
Yes	6	255	2.35	8	198	4.04	2.46 (0.64–9.49)	4	257	1.56	0.83 (0.17–3.97)	2	174	1.15	0.68 (0.09–4.69)

PY, person-years; IR, incidence rate per 100 person-years; HR, hazard ratio; CI, confidence interval; ^†^ HR adjusted for age, sex, comorbidities, NIHSS score, hospital level, urbanization level, and residential area of Taiwan; * *p* < 0.05, ** *p* < 0.01, *** *p* < 0.001; – Unable to calculate because there are few or no events in rehabilitation group and comparison cohort.

**Table 4:** Cox regression analyses of age, sex, and NIHSS score associated with pressure ulcer for the rehabilitation cohort by rehabilitation intensity.

	Rehabilitation Intensity												
	1–3 Times (*n* = 5066)			4–14 Times (*n* = 3489)					≥15 Times (*n* = 1274)				
Rehabilitation Cohort	Pressure Ulcer	PY	IR	Pressure Ulcer	PY	IR	Crude HR (95%CI)	Adjusted HR ^†^ (95%CI)	Pressure Ulcer	PY	IR	Crude HR (95%CI)	Adjusted HR ^†^ (95%CI)
	Event			Event					Event				
Age													
<65	113	10,147	1.11	97	7820	1.24	1.11 (0.85–1.46)	0.96 (0.72–1.27)	41	3270	1.25	1.13 (0.79–1.61)	0.91 (0.63–1.32)
≥65	454	13,257	3.42	304	8871	3.43	1.00 (0.86–1.16)	1.12 (0.85–1.47)	71	3104	2.29	0.67 (0.52–0.86) **	1.14 (0.79–1.63)
Sex													
Female	247	9517	2.6	166	6684	2.48	0.96 (0.78–1.17)	1.04 (0.85–1.27)	41	2739	1.5	0.58 (0.41–0.81) **	0.67 (0.48–0.94) *
Male	320	13,887	2.3	235	10,007	2.35	1.02 (0.86–1.20)	1.06 (0.90–1.26)	71	3635	1.95	0.84 (0.65–1.09)	0.94 (0.73–1.22)
NIHSS score													
≤13	389	19,033	2.04	247	11,178	2.21	1.08 (0.92–1.27)	1.08 (0.92–1.27)	68	4195	1.62	0.79 (0.61–1.02)	0.86 (0.66–1.12)
>13	178	4371	4.07	154	5513	2.79	0.71 (0.57–0.89) **	0.84 (0.67–1.04)	44	2179	2.02	0.52 (0.37–0.72) ***	0.63 (0.45–0.88) **

PY, person-years; IR, incidence rate per 100 person-years; HR, hazard ratio; CI, confidence interval; ^†^ HR adjusted for age, sex, and level of rehab intensity; * *p* < 0.05, ** *p* < 0.01, *** *p* < 0.001.

## Data Availability

All relevant data are within the paper.
